# Interferon alpha promotes caspase-8 dependent ultraviolet light-mediated keratinocyte apoptosis via interferon regulatory factor 1

**DOI:** 10.3389/fimmu.2024.1384606

**Published:** 2024-04-10

**Authors:** Shannon N. Loftus, Mehrnaz Gharaee-Kermani, Bin Xu, Tyson M. Moore, Andrew Hannoudi, Mischa J. Mallbris, Benjamin Klein, Johann E. Gudjonsson, J. Michelle Kahlenberg

**Affiliations:** ^1^Division of Rheumatology, Department of Internal Medicine, University of Michigan, Ann Arbor, MI, United States; ^2^Graduate Program in Immunology, University of Michigan, Ann Arbor, MI, United States; ^3^Department of Dermatology, University of Michigan, Ann Arbor, MI, United States

**Keywords:** interferon, apoptosis, ultraviolet light, keratinocyte, lupus

## Abstract

**Introduction:**

Ultraviolet (UV) light is a known trigger of both cutaneous and systemic disease manifestations in lupus patients. Lupus skin has elevated expression of type I interferons (IFNs) that promote increased keratinocyte (KC) death after UV exposure. The mechanisms by which KC cell death is increased by type I IFNs are unknown.

**Methods:**

Here, we examine the specific cell death pathways that are activated in KCs by type I IFN priming and UVB exposure using a variety of pharmacological and genetic approaches. Mice that overexpress *Ifnk* in the epidermis were exposed to UVB light and cell death was measured. RNA-sequencing from IFN-treated KCs was analyzed to identify candidate genes for further analysis that could drive enhanced cell death responses after UVB exposure.

**Results:**

We identify enhanced activation of caspase-8 dependent apoptosis, but not other cell death pathways, in type I IFN and UVB-exposed KCs. *In vivo*, overexpression of epidermal *Ifnk* resulted in increased apoptosis in murine skin after UVB treatment. This increase in KC apoptosis was not dependent on known death ligands but rather dependent on type I IFN-upregulation of interferon regulatory factor 1 (IRF1).

**Discussion:**

These data suggest that enhanced sensitivity to UV light exhibited by lupus patients results from type I IFN priming of KCs that drives IRF1 expression resulting in caspase-8 activation and increased apoptosis after minimal exposures to UVB.

## 1 Introduction

Systemic lupus erythematosus (SLE) is an autoimmune disease with frequent skin involvement, termed cutaneous lupus erythematosus (CLE). A characteristic feature of SLE and CLE is photosensitivity, affecting up to 93% of patients (1) and triggering cutaneous or systemic disease flares. The mechanisms driving these responses are not well understood.

In healthy skin, exposure to UV light generates an immunosuppressive environment that limits severe tissue injury (2). However, in SLE skin, exposure to UVB radiation results in overactivation of the immune system that induces autoimmune responses and lesion development (3, 4). While mechanisms underlying these differences are likely multifactorial, studies suggest an important role for type I IFNs in driving these differential responses.

Type I IFNs are implicated in SLE and CLE pathogenesis and progression as well as keratinocyte (KC) death. Expression of type I IFN response genes is increased in lesional and non-lesional KCs from lupus patients vs. healthy controls (5, 6). Moreover, cutaneous type I IFN production from both KCs and infiltrating immune cells is increased after UVB (7–9). Importantly, KC secretion of IFNs is more significantly increased in lupus KCs vs. controls after UVB treatment (8, 10). While short bursts of type I IFNs are protective against UVB-induced inflammation in healthy skin (7), they seem to be more pathogenic in SLE skin where they are chronically present and promote pro-inflammatory cytokine secretion and immune cell recruitment and activation (8, 11, 12). Further, we have previously demonstrated that SLE KCs exhibit increased UVB-induced death vs. healthy controls that is dependent on IFN signaling (13). However, the manner in which type I IFNs regulate this enhanced cell death phenotype in KCs is unknown.

In this paper, we investigate the role of type I IFNs in activation and regulation of cell death pathways in UVB-irradiated KCs. We show that type I IFNs promote enhanced UVB-driven apoptosis of KCs rather than inflammatory forms of cell death. Further, we demonstrate that this is a caspase-8-dependent process that occurs in the absence of death-ligand signaling. Lastly, we show evidence that increased rates of apoptosis require IFN-induced upregulation of interferon regulatory factor 1 (IRF1) in KCs, a known modulator of caspase-8 (14). Thus, our work identifies IFN-enhanced apoptosis, and not inflammatory forms of cell death, as a critical step in driving photosensitivity in SLE.

## 2 Materials and methods

### 2.1 Cell culture

N/TERTs (N/TERT-2G) (15), an immortalized human KC line, were used with permission from James G. Rheinwald for *in vitro* assays. Cells were grown in serum-free KC growth medium (supplemented with 30µg/mL bovine pituitary extract, 0.2ng/mL epidermal growth factor, 0.3mM calcium chloride) as previously described (Gibco, Grand Island, NY) (13). N/TERTs were maintained at 37°C with 5% CO2.

Primary human KCs were isolated from skin biopsies from healthy controls as previously described (8). KCs were cultured in serum-free KC growth medium (EpiLife with human keratinocyte growth supplement) and passaged at 60% confluency to avoid differentiation. Culture medium was replaced every 2-3 days and purity was assessed by morphology.

### 2.2 Mice

10-14 week old female wild-type C57BL/6 mice were purchased from Charles River Laboratories. Mice overexpressing *Ifnk* under the keratin 14 promoter (to promote epidermal overexpression) on the C57BL/6 background were generated as previously described (16). Mice were housed in specific pathogen-free facilities in the Unit for Animal Laboratory Medicine facility at the University of Michigan. All animal procedures were performed in accordance with University of Michigan Institutional Animal Care and Use Committee-approved protocols.

### 2.3 UVB irradiation

Mouse dorsal fur was removed via shaving and depilation with Veet (Reckitt Benckiser, UK). Mice were placed in restrainers with facial protection and treated once ±250mJ/cm^2^ UVB light using the UV-2 ultraviolet irradiation system (Tyler Research). UVB light was provided by cascade-phosphor ultraviolet generators that emit 310nm UVB radiation.

For irradiation of cultured cells at approximately 80% confluence, growth medium was replaced with PBS and cells were treated with 0-50mJ/cm^2^ UVB light as indicated in specific experiments. PBS was removed immediately following irradiation and replaced with growth medium. Cells were incubated at 37°C for the indicated times.

Inhibitors of cell death (Ac-YVAD-cmk, Sigma Aldrich SML0429; Necrostatin-1, Cayman Chemical 11658; GSK’872, Tocris Bioscience 64-921-0; Z-IETD-FMK, Selleckchem S7314; Z-LEHD-FMK, Selleckchem S7313; Z-VAD-FMK, Selleckchem S7023; UAMC-3203, Selleckchem S8972) were added 30 minutes prior to IFN treatment and again immediately following UVB exposure.

For neutralizing antibody experiments, N/TERTs were treated with isotype control (R&D MAB002, MAB0041) or neutralizing antibodies targeting TRAIL (100ng/ml; R&D MAB375), TNF-α (5µg/ml; R&D MAB210) or FasL (1µg/ml; R&D MAB126) immediately following UVB exposure.

### 2.4 Flow cytometry

N/TERTs were treated ±1000U/ml IFN-α (Intron A, interferon alfa-2b, Merck) for 16 hours then treated ±50mJ/cm^2^ UVB light. Four hours later, cells were trypsinized and pelleted with supernatant to ensure no loss of detached dying cells.

Annexin V (AV) and propidium iodide (PI) staining: Cells were washed twice in flow block (1% bovine serum albumin and 1% horse serum in PBS). 100,000 cells were stained with FITC-AV and PI (Biolegend, San Diego, CA) for 15 minutes. Flow cytometry data was collected via BD LSR II flow cytometer and analyzed using FlowJo V10.

Cleaved caspase-3 staining: Cells were washed twice in PBS. Approximately 500,000 cells were stained with Fixable Viability Dye eFluor™ 780 (Invitrogen, Waltham, MA) for 30 minutes then fixed with 4% paraformaldehyde for 20 minutes. Following fixation, cells were washed in IFA-Tx buffer (4% FBS, 0.1% Triton X-100, 100mM HEPES pH 7.4, in 0.9% sodium chloride) then stained with anti-cleaved caspase-3 (1:500; #9661 or 9664, Cell Signaling Technology, Danver, MA) for 1 hour at 4°C and Alexa Fluor 488-conjugated donkey anti-rabbit IgG (1:200; Invitrogen) for 1 hour at 4°C. Flow cytometry data was collected as described above.

### 2.5 RNA isolation and quantitative reverse-transcription PCR

Total RNA was isolated from cells using Direct-Zol RNA MiniPrep kit (Zymo Research, Irvine, CA) or RNeasy Plus Mini Kit (Qiagen, Germantown, MD) following manufacturers’ instructions. 200ng RNA was converted to cDNA using iScript™ cDNA Synthesis Kit (Bio-Rad, Hercules, California). qRT-PCR was performed in technical triplicates using PowerUp™ SYBR™ Green Master Mix (Applied Biosystems, Waltham, Massachusetts), on either QuantStudio or ABI Prism 7900HT Real-Time qPCR Systems (Applied Biosystems) with the assistance of the University of Michigan Advanced Genomics Core. The primers used were as follows (all listed 5’ ➔ 3’): *CASP8* (forward, AGAAGAGGGTCATCCTGGGAGA; reverse, TCAGGACTTCCTTCAAGGCTGC), *IRF1* (forward, CTGTGCGAGTGTACCGGATG; reverse, ATCCCCACATGACTTCCTCTT), *TNFSF10* (forward, TGCGTGCTGATCGTGATCTTC; reverse, GCTCGTTGGTAAAGTACACGTA), *XAF1* (forward, GAGCGCCCTGTTGAGTGTAA; reverse, CACAGTAGGACTCGTGGAGC), *GAPDH* (forward, CTGGGCTACACTGAGCACC; reverse, AAGTGGTCGTTGAGGGCAATG), *BACTIN* (forward, CCTCGCCTTTGCCGATCC; reverse, GCGCGGCGATATCATCATCC). Gene expression was normalized to β-actin or GAPDH and relative fold change compared to control was calculated using the comparative 2^-ΔΔCT^ method.

### 2.6 Gene knockdown by siRNA

For gene knockdown, Accell SMARTpool siRNAs were used as previously described (17). Briefly, N/TERTs were incubated with 1µM Accell SMARTpool siRNA targeting *IRF1* or *CASP8* (Dharmacon #E-011704-00, E-003466-00) for 48 hours. Accell Non-targeting Control Pool siRNA (Dharmacon #D-001910-10) was used as a negative control.

### 2.7 Immunofluorescence

For detection of cleaved caspase-3/7 in N/TERTs, cultured cells were treated as indicated and incubated with 5µM CellEvent Caspase-3/7 Green detection reagent (Invitrogen) following UVB exposure. Cells were counterstained with 0.5% Hoechst and images acquired using a Zeiss microscope. Percent cleaved caspase-3/7+ cells were quantified by manually counting the # of cells positive for cleaved caspase-3/7 (green)/total # of cells positive for Hoechst (blue) averaged for three 20x fields of view.

For detection of cleaved caspase-8 in N/TERTs, cultured cells were treated as indicated, fixed with 4% paraformaldehyde, permeabilized with 0.1% Triton-100 in PBS, and blocked with 10% goat serum and 5% BSA for 30 minutes at room temperature. Cells were incubated with cleaved caspase-8 antibody (1:100 dilution; #98134 Cell Signaling Technology) for 1 hour at room temperature, washed, incubated with Alexa Fluor 488-conjugated goat anti-rabbit IgG secondary antibody (1:100 dilution; #A-11008 Invitrogen) for 30 minutes at room temperature, washed, and counterstained with 0.5% Hoechst. Images were acquired using a Zeiss microscope at indicated magnifications. Percent cleaved caspase-8^+^ cells was quantified by manually counting the # of cells positive for cleaved-caspase-8 (green)/total # of cells positive for Hoechst (blue).

### 2.8 TUNEL staining

For TUNEL staining in mouse skin, formalin-fixed, paraffin-embedded sections were utilized, and staining was performed according to the manufacturer’s protocol (Sigma-Aldrich). Briefly, slides were dewaxed, rehydrated, and incubated with Proteinase K solution. Slides were treated with TUNEL reaction mixture in a humidified chamber, washed in PBS, and mounted with VECTASHIELD Mounting Medium with DAPI (Vector Laboratories). Images were acquired using a Zeiss microscope at indicated magnifications. Percent epidermal TUNEL^+^ cells was quantified by manually counting the # cells positive for TUNEL (red)/# of cells positive for DAPI (blue) in a defined region of interest (ROI) in the epidermis.

### 2.9 Immunohistochemistry

For detection of cleaved caspase-3 in mouse skin, formalin-fixed, paraffin-embedded sections were heated at 60°C for 1 hour, deparaffinized, rehydrated, and heated at 100°C for 20 minutes in Tris-EDTA buffer (pH 9.0) for antigen retrieval. Slides were washed, treated with 3% hydrogen peroxide in PBS for 5 minutes, blocked in goat serum for 1 hour, and incubated with Human/Mouse Cleaved Caspase-3 (Asp175) antibody (1:100 dilution, MAB835 R&D) overnight at 4°C. Isotype controls (#3900; Cell Signaling Technology) were stained in parallel with each set of slides. All slides were incubated with biotinylated goat anti-rabbit IgG secondary antibody (1:200; Vector Laboratories, Newark, California), followed by incubation with VECTASTAIN Elite ABC reagent (Vector Laboratories) and detection with 3,3’-diaminobenzidine under a light microscope. Slides were counterstained with hematoxylin, dehydrated, and mounted. Images were acquired using a Zeiss microscope at indicated magnifications. Percent epidermal caspase-3^+^ cells was quantified by manually counting the # of positive cells (brown)/total # of nuclei in the epidermis averaged for three 20x fields of view.

### 2.10 ELISA

Supernatants from cultured N/TERTs were assayed for TNF-α or IL-1β using the TNF alpha Human ELISA Kit (Invitrogen) or the Human IL-1 beta Quantikine ELISA Kit (R&D), respectively, following the manufacturer’s instructions.

### 2.11 Cell viability assay

For ferroptosis experiments, cell viability was measured by Cell Counting Kit 8 (WST-8) (Abcam) according to the manufacturer’s protocol.

### 2.12 Western blot

Total protein was isolated from cultured KCs and cellular supernatants using RIPA buffer supplemented with protease and phosphatase inhibitors. Protein concentration was measured by Pierce BCA Protein Assay Kit (Thermo Scientific, Waltham, MA) per manufacturer’s directions. 10-25ug protein was run on 4-12% precast polyacrylamide gels (Invitrogen) and transferred to 0.45µm polyvinylidene difluoride membranes. Membranes were blocked with 5% nonfat dry milk or BSA and incubated overnight at 4°C with primary antibodies (1:1000 dilution; XAF1 #13805; TRAIL #3219; IRF-1 #8478; Phospho-MLKL (Ser358) #91689; MLKL #14993; Cell Signaling Technology) followed by HRP-conjugated goat anti-rabbit IgG (1:10,000 dilution; sc-2301 Santa Cruz, Dallas, TX) or HRP-conjugated mouse anti-rabbit IgG (1:2000 dilution; sc-2357 Santa Cruz). Protein bands were detected by chemiluminescence using SuperSignal West Dura Extended Duration Substrate (Thermo Scientific) and imaged by Omega Lum C (Gel Company, San Francisco, CA). Quantification was completed with ImageJ software relative to β-actin loading control (1:1000 dilution; #4967 Cell Signaling Technology).

### 2.13 Generation of stable knockdown lines using viral shRNAs

Knockdown of XAF1 in N/TERTs was performed by transduction with lentivirus expressing two different shRNAs targeting human XAF1 or an shRNA control (shCTL). Two different lentivirus-based plasmids of Mission shRNA (clone numbers TRCN0000426403 and TRCN0000134449) against human XAF1 and the shCTL vector TRC2 pLKO.5-puro non-mammalian shRNA (sHC202) were obtained from Sigma-Aldrich (Burlington, MA). 293T cells were co-transfected with the shRNA of interest and packaging plasmids psPAX2 and pMD2 by the Lipofectamine 2000 (Invitrogen) method in OptiMEM (Gibco). Six hours following transfection, media was replaced with fresh KC growth medium. Twenty-four hours post-transfection, media containing lentivirus was passed through 0.45µm filters, supplemented with 8µg/ml polybrene, and used to transduce sub-confluent N/TERTs. Fresh KC growth medium was added to the 293T cells and this process was repeated 8 and 24 hours later. The shRNA- and shCTL-transduced N/TERTs were selected for using 10-12µg/ml puromycin and cells were maintained in 8µg/ml puromycin until experiment.

### 2.14 Statistical analysis

All data graphed and statistics performed using GraphPad Prism 9. Data are presented as mean ± SEM. For comparisons between two groups, paired or unpaired two-tailed t tests were used for normally distributed data and Mann-Whitney or Wilcoxon matched-pairs signed rank tests were used for non-normally distributed data. P-values <0.05 were considered statistically significant.

## 3 Results

### 3.1 Type I IFNs promote KC apoptosis after UVB exposure

**Figure 1:**
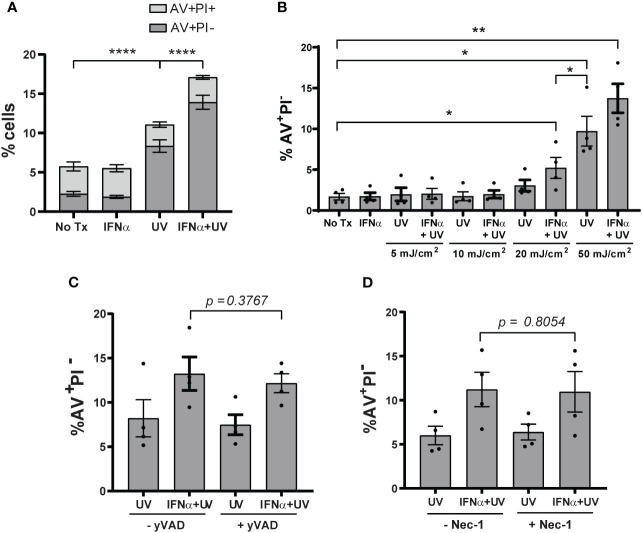
Type I IFNs promote KC apoptosis after UVB exposure. N/TERTs were treated with or without 1000U/ml IFN-α for 16 hours followed by exposure to **(A)** 50mJ/cm² UVB (*n*=17 independent experiments) or **(B)** 0-50mJ/cm² (*n*=4 independent experiments). Cell death response assessed four hours after UVB exposure by Annexin V (AV) and propidium iodide (PI) staining by flow cytometry. **(C, D)** N/TERTs were pretreated with or without 10µM **(C)** caspase-1-inhibitor Ac-YVAD-cmk (yVAD) or **(D)** RIPK1-inhibitor Necrostatin-1 (Nec-1) prior to IFN-α and UVB stimulation and AV/PI staining as before (n=3-4 independent experiments). Each dot represents the average of duplicate conditions for each individual experiment. Data analyzed by paired *t* tests or Wilcoxon matched-pairs signed rank tests. *<0.05, **<0.01, ****<0.0001.

Type I IFNs are implicated in increasing KC cell death after exposure to UVB light (13). We first sought to confirm this by treating N/TERTs, an immortalized human KC line, with IFN-α prior to exposure to UVB. To examine cell death, we used flow cytometry and defined cells likely to be apoptotic as Annexin V^+^ Propidium Iodide^-^ (AV^+^PI^-^) and defined cells likely undergoing other forms of cell death in which membrane permeability is disrupted as AV^+^PI^+^ (**Supplementary Figure S1A**). Four hours after UVB (the earliest timepoint at which a difference in cell death was detected), the percentage of AV^+^PI^-^ and not AV^+^PI^+^ cells increased significantly with UVB alone and this was further increased by IFN-α priming (**Figure 1A**). These results confirmed that type I IFNs promote enhanced KC death after UVB and suggests this may occur through activation of apoptotic pathways.

As the dose of UV light needed to cause erythema in SLE patients is lower compared to healthy controls (18), we then examined whether type I IFN priming of N/TERTs could lower the dose of UV needed to increase cell death. As expected, priming with IFN-α lowered the dose of UVB required to significantly increase the percentage of AV^+^PI^-^ cells (**Figure 1B**). This suggests that the ability of IFN-α to increase UVB-driven death *in vitro* mimics what is observed clinically in SLE patients with lower MEDs. We thus chose 50mJ/cm^2^ as the dose to study as it was the lowest dose that provided both for detection of cell death by UV treatment and type I IFN-mediated enhancement of this process.

Several cell death pathways can be activated by UVB irradiation (19, 20). Because pyroptosis, necroptosis, and ferroptosis are inflammatory forms of cell death, we hypothesized that increased activation of these pathways by IFN priming could be a factor in UVB-induced inflammation in lupus skin. To test this, we used small-molecule inhibitors of caspase-1 (Ac-YVAD-cmk) to block pyroptosis, of receptor-interacting serine/threonine-protein kinase-1 (RIPK1; Necrostatin-1) and RIPK3 (GSK’872) to block necroptosis, and UAMC-3202 to block ferroptosis. All inhibitors were able to block their respective forms of cell death (**Supplementary Figures S1B–E, G**). To test their effects on UV-mediated cell death, N/TERTs were pretreated with individual inhibitors and then stimulated with IFN-α and UVB. Inhibition of these pathways had no effect on the percentage of AV^+^PI^-^ cells (**Figures 1C, D**; **Supplementary Figure S1F**) or cell viability (**Supplementary Figure S1H**). Together, these results suggest that type I IFN priming sensitizes KCs to increased UVB-induced apoptosis and not pyroptosis, necroptosis, or ferroptosis.

### 3.2 Type I IFN-priming enhances caspase-8 driven extrinsic apoptosis in UVB-irradiated KCs

**Figure 2:**
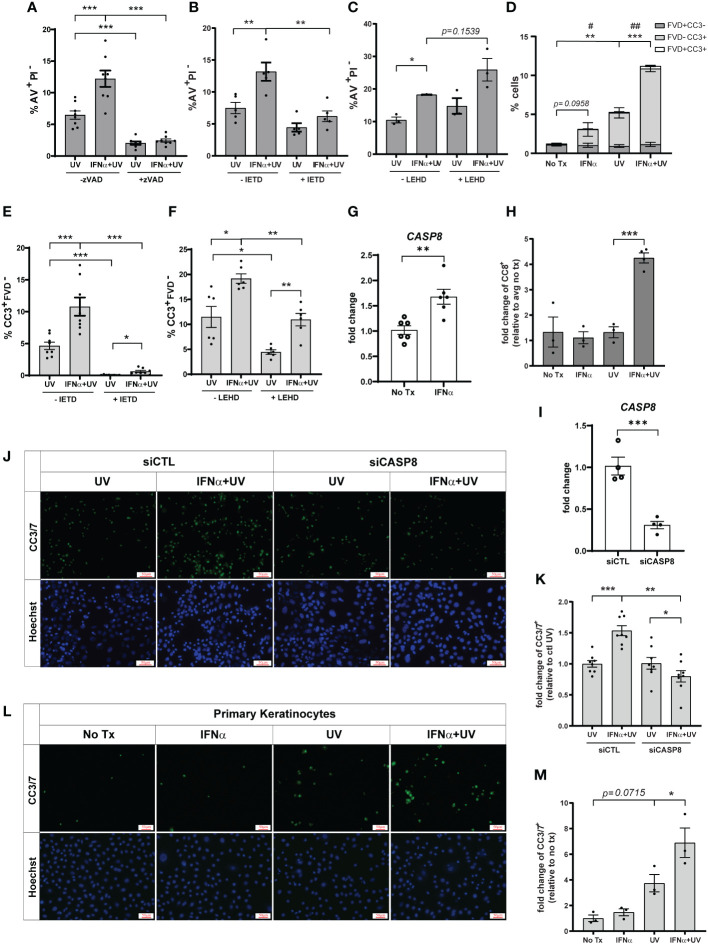
Type I IFNs promote caspase-8 mediated KC apoptosis after UVB exposure. N/TERTs were pretreated with or without 10µM **(A)** pan-caspase-inhibitor Z-VAD-FMK (zVAD), **(B, E)** caspase-8-inhibitor Z-IETD-FMK (IETD), or **(C, F)** caspase-9-inhibitor Z-LEHD-FMK (LEHD), treated with or without 1000U/ml IFN-α for 16 hours, then exposed to 50mJ/cm² UVB. Cell death response assessed four hours after UVB exposure by staining for **(A–C)** Annexin V (AV) and propidium iodide (PI) or **(D-F)** cleaved caspase-3 (CC3) and fixable viability dye (FVD) and flow cytometry (*n*=3-8 independent experiments, each dot represents the average of duplicate conditions for each individual experiment). **(G)** Fold change expression of *CASP8* in N/TERTs treated with or without 1000U/ml IFN-α for 16 hours, assessed by qRT-PCR (*n*=6). **(H)** Fold change expression of cleaved caspase-8 (CC8) in N/TERTs treated with IFN-α and UVB as before, assessed by immunofluorescence (n=3-4). Scale bar: 50µm. **(I–K)** N/TERTs were treated with scrambled (siCTL) or caspase-8 targeting (siCASP8) siRNA. **(I)** Fold change expression of *CASP8* at baseline was assessed by qRT-PCR (*n*=4 replicates per condition from 2 independent experiments). **(J–M)** Representative images and quantification of cleaved caspase-3/7 in **(J, K)** siRNA-treated N/TERTs (*n*=8 replicates per condition from 2 independent experiments) or **(L, M)** primary keratinocytes following IFN-α and UVB stimulation (*n*=3 independent experiments, each dot represents the average of quadruplicate conditions for each individual experiment). Scale bar: 50µm. Data analyzed by **(A–F, K, M)** paired or **(G, I)** unpaired *t* tests. **(D)** *p*-values designated by asterisks (*) correspond to FVD^-^CC3^+^. p-values designated by number signs (^#^) correspond to FVD^+^CC3^-^. *, ^#^<0.05, **,^##^<0.01, ***<0.001.

Apoptosis is generally mediated by activation of caspases; however, caspase-independent apoptotic pathways exist (21). To determine the requirement for caspases in type I IFN and UVB-regulated apoptosis, we pretreated cells with Z-VAD-FMK, a pan-caspase-inhibitor, prior to IFN-α and UVB stimulation. IFN-α-induced upregulation of UVB-mediated apoptosis was entirely abrogated by pan-caspase inhibition (**Figure 2A**). UVB-mediated apoptosis typically occurs through the caspase-9-driven intrinsic pathway or the caspase-8-driven extrinsic pathway (22–24). Therefore, we next examined dependence on these pathways in IFN-primed KCs following UVB. To do so, we pretreated cells with caspase-8 inhibitor Z-IETD-FMK, or caspase-9 inhibitor Z-LEHD-FMK, prior to IFN-α and UVB stimulation. Intriguingly, only caspase-8 inhibition significantly reduced AV^+^PI^-^ cells following combination treatment with IFN-α and UVB (**Figure 2B**), while caspase-9 inhibition had no effect on this population (**Figure 2C**).

As a secondary means for confirming the effect of caspase-8 inhibition on apoptosis we examined caspase-3 cleavage, which occurs downstream of caspase-8 activation. We stained cells with anti-cleaved caspase-3 (CC3) and a fixable viability dye (FVD) to differentiate between early (CC3^+^FVD^-^) and late apoptosis (CC3^+^FVD^+^) (**Supplementary Figure S2A**). We found that, as expected, UVB exposure alone significantly increased CC3^+^FVD^-^ cells, and IFN-α priming prior to UVB further increased this population (**Figure 2D**). In agreement with the AV staining, inhibition of caspase-8 almost completely abrogated UVB-mediated and IFN-enhanced apoptosis as measured by CC3^+^FVD^-^ (**Figure 2E**). Inhibition of caspase-9 did significantly decrease the percentage of CC3^+^FVD^-^ cells after UVB treatment, but the effect on IFN-mediated enhancement was modest (**Figure 2F**). We further showed that IFN-α significantly increased expression of caspase-8 in N/TERTs (**Figure 2G**) while combination treatment with IFN-α and UVB increased caspase-8 cleavage and, thus, activation (**Figure 2H**; **Supplementary Figure S2B**).

To complement our pharmacological studies and to account for any non-specificity of the caspase inhibitors, we used a genetic approach to confirm caspase-8 dependence. Accell siRNA was used to knockdown expression of caspase-8 in N/TERTs (siCASP8) relative to a non-targeting control line (**Figure 2I**). siCASP8 and non-targeting control cells were treated with IFN-α and UVB and caspase-3 cleavage was assessed. Here, we used fluorescent microscopy to detect cells with activated caspase-3 as insufficient cell numbers are obtained with Accell siRNA knockdown for flow cytometry. While slightly blunted, IFN priming still enhanced caspase-3 activation compared to UV treatment alone in the presence of non-targeting control siRNA (**Supplementary Figure S2C**). Importantly, in agreement with the inhibitor data, loss of caspase-8 significantly reduced the percentage of CC3^+^ cells following UVB exposure and eliminated the IFN-enhancement of apoptosis (**Figures 2J, K**).

Lastly, we showed that IFN priming significantly increased caspase-3 activation following UVB exposure in primary keratinocytes from healthy donors, suggesting relevance for this phenotype in human skin (**Figures 2L, M**). Together, these data suggest that type I IFN priming drives an enhanced apoptotic phenotype in KCs following exposure to UVB that depends on activation of caspase-8.

### 3.3 Epidermal overexpression of IFN-κ increases UVB-induced apoptosis *in vivo*

**Figure 3:**
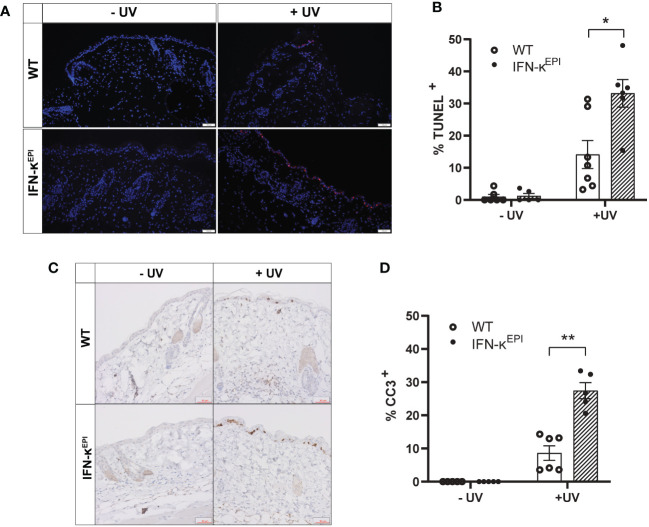
Epidermal overexpression of IFN-к increases UVB-induced apoptosis *in vivo*. Mice that overexpress IFN-к in the epidermis under the control of the keratin-14 promoter (IFN-к^EPI^) and C57BL/6 wild-type (WT) mice were treated with or without 250mJ/cm² UVB (*n*=5-7 mice per group). Levels of apoptosis examined 24 hours later via **(A, B)** TUNEL assays (scale bar: 100µm) or **(C, D)** cleaved caspase-3 (CC3) immunostaining (scale bar: 50µm). Each symbol represents an individual mouse. Data analyzed by Mann-Whitney test. *<0.05, **<0.01.

To investigate how modulation of type I IFNs affects UVB-induced apoptosis *in vivo*, we used mice that overexpress IFN-kappa (IFN-κ) 2-4 fold above normal levels in the epidermis (IFN-κ^EPI^ mice) (**Supplementary Figure S3**) (16, 25). C57BL/6 (wild-type) and IFN-κ^EPI^ mice were treated with or without UVB on their dorsum. Levels of epidermal apoptosis were examined 24 hours following stimulation with 250mJ/cm^2^ UVB via TUNEL assays and immunostaining for cleaved caspase-3. Consistent with our cell culture data, we detected no difference in baseline levels of apoptosis between wild-type and IFN-κ^EPI^ mice (**Figures 3A–D**). However, following acute UVB exposure, IFN-κ^EPI^ mice had significantly elevated levels of TUNEL (**Figures 3A, B**) and cleaved caspase-3 staining (**Figures 3C, D**). These data support a role for type I IFNs in increasing UVB-induced apoptosis *in vivo*.

### 3.4 Type I IFN-enhanced apoptosis after UVB exposure is XAF1-independent

To better understand the mechanism by which IFNs influence KC responses to UVB, we examined RNA-sequencing (RNA-seq) analysis from non-lesional SLE skin and healthy control (HC) skin treated ± IFN-α as previously reported (17). The pro-apoptotic gene X-linked inhibitor of apoptosis (XIAP)-associated factor 1 (*XAF1*) was highly upregulated by IFN-α treatment in HC KCs, and even more so in SLE KCs (**Supplementary Figure S4A**). XAF1 can enhance apoptosis through a variety of mechanisms (26). Thus, we hypothesized that XAF1 upregulation by type I IFNs renders KCs more sensitive to UVB-induced apoptosis.

**Figure 4:**
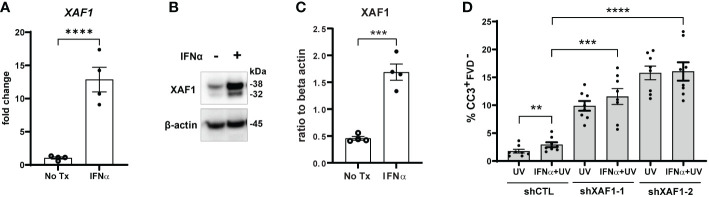
Type I IFN-enhanced apoptosis after UVB exposure is XAF1-indpendent. **(A–C)** N/TERTs were treated with or without 1000U/ml IFN-α for 16 hours and **(A)** fold change expression of *XAF1* was assessed by qRT-PCR (*n*=4) **(B, C)** and protein levels of XAF1 were assessed by Western blot (*n*=4 from 2 independent experiments). **(D)** Stable XAF1-knockdown N/TERTs were generated by transducing N/TERTs with lentivirus expressing shRNAs targeting XAF1 (shXAF1-1, -2) or control shRNA (shCTL). Cells were treated with or without 1000U/ml IFN-α for 16 hours followed by exposure to 50mJ/cm² UVB. Cleaved caspase-3 (CC3) and fixable viability dye (FVD) staining was measured four hours later by flow cytometry (*n*=8 replicates from 4 independent experiments). Data analyzed by **(A, C)** unpaired or **(D)** paired *t* tests. **<0.01, ***<0.001, ****<0.0001.

To test this, we first confirmed that IFN-α treatment of N/TERTs increased expression of XAF1 mRNA (**Figure 4A**) and protein (**Figures 4B, C**). We next generated two stable XAF1-knockdown N/TERT lines by transducing N/TERTs with lentivirus expressing shRNA targeting *XAF1* (shXAF1-1 and -2) or a control shRNA (shCTL). Knockdown efficacy of XAF1 was confirmed by Western blot (**Supplementary Figures S4B, C**). shXAF1 N/TERTs were stimulated with IFN-α and UVB as before and caspase-3 cleavage was assessed. Surprisingly, loss of XAF1 resulted in a robust increase in UVB-induced apoptosis compared to control (**Figure 4D**). No enhancement of apoptosis was seen with IFN-α treatment in the shXAF1 lines; however, given the robust increase in baseline apoptosis, it is hard to interpret the interaction of XAF1 and IFN signaling in this context. Therefore, our conclusion is that XAF1 may serve as a negative regulator of cell death after UVB.

### 3.5 Type I IFN-enhanced apoptosis after UVB exposure is death ligand-independent

Cutaneous disease in murine lupus models is, in part, driven by members of the tumor necrosis factor (TNF) and TNF-receptor (TNFR) superfamilies (27, 28). We, therefore, examined our RNA-seq data to determine if differential regulation of these pro-apoptotic genes by IFN-α could promote UVB-driven apoptosis in our system. TNFR superfamily member 12A (*TNFRSF12A*; encodes Fn14) was slightly upregulated by IFN-α treatment in the SLE KCs only, however, there was no change in TNF superfamily member 12 (*TNFSF12*; encodes TWEAK) expression in HC or SLE KCs (**Supplementary Figure S4A**). Further, while *FAS* was moderately increased by IFN-α treatment in the SLE KCs only, *FASLG* (encodes FasL) expression was not detected in these samples (**Supplementary Figure S4A**). Thus, these pathways did not exhibit differences that would explain IFN-enhanced apoptotic changes. However, our RNA-seq did identify differential expression of other TNF/TNFR superfamily members that can induce caspase-8-dependent apoptosis, as well as increased expression of *CASP8* itself in response to IFN treatment in both HC and SLE KCs. Specifically, *TNFSF10* [encodes TNF-related apoptosis-inducing ligand (TRAIL)] and *TNFRSF1B* (encodes TNFR2) were upregulated in IFN-α treated KCs and more so in SLE vs. HCs (**Supplementary Figure S4A**). Thus, we hypothesized that IFN-induced alterations in TRAIL and/or TNF-α signaling mediate increased apoptosis of KCs after UVB.

In agreement with the RNA-seq, N/TERTs stimulated with IFN-α had increased expression of TRAIL mRNA (**Supplementary Figure S5A**) and protein (**Supplementary Figures S5B, C**). As our analysis only identified differences in receptor expression and not TNF-α itself, we confirmed that N/TERTs secrete TNF-α following UVB (**Supplementary Figure S5D**). There was a small, but significant increase in TNF-α secretion with IFN priming prior to UVB, suggesting that IFN-induced changes in TNF-receptor expression paired with increased cytokine secretion after UVB may drive increased signaling. To determine whether increased TRAIL or TNF-α signaling was responsible for enhanced apoptosis, we used neutralizing antibodies against TRAIL and TNF-α. We first confirmed that treatment with these antibodies prevented apoptosis in cells treated with recombinant TRAIL or TNF-α (**Supplementary Figures S5E, F**). N/TERTs were treated with neutralizing TRAIL or TNF-α antibodies or isotype control following stimulation with IFN-α and UVB. Surprisingly, inhibition of neither TRAIL nor TNF-α influenced IFN-enhanced apoptosis after UVB (**Supplementary Figures S5G, H**). To account for any compensatory effect, we treated N/TERTs with both anti-TRAIL and anti-TNF-α. Again, we saw no change in levels of apoptosis suggesting no involvement of these ligands (**Supplementary Figure S5I**). We also confirmed that FasL inhibition had no effect on IFN-α and UVB induced apoptosis (**Supplementary Figure S5J**). Together, these data suggest that type I IFN-enhanced apoptosis after UVB exposure occurs independently of several known death ligands.

### 3.6 IRF1 is upregulated in KCs by type I IFN treatment and drives enhanced UVB-driven apoptosis

**Figure 5:**
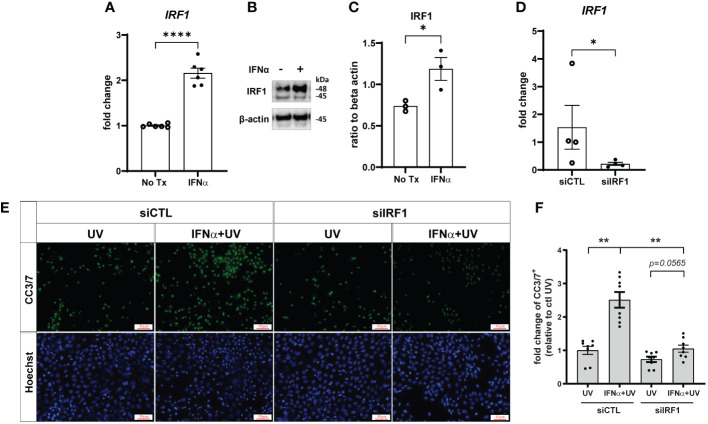
Type I IFN-enhanced apoptosis after UVB exposure is IRF1-dependent. N/TERTs were treated with or without 1000U/ml IFN-α for 16 hours and **(A)** fold change expression of *IRF1* was assessed by qRT-PCR (*n*=6) and **(B, C)** protein levels of IRF1 were assessed by Western blot (*n*=3). **(D–F)** N/TERTs were treated with scrambled (siCTL) or IRF1 targeting (siIRF1) siRNA. **(D)** Fold change expression of *IRF1* at baseline was assessed by qRT-PCR (*n*=4). **(E, F)** Representative images and quantification of cleaved caspase-3/7 in siRNA-treated N/TERTs following IFN-α and 50mJ/cm^2^ UVB stimulation (*n*=8 replicates per condition from 2 independent experiments). Scale bar: 50µm. Data analyzed by **(A, C, D)** unpaired *t* tests or **(F)** paired *t* tests and Wilcoxon matched-pairs signed rank test. *<0.05, **<0.01, ****<0.0001.

IRF1 is an IFN-inducible transcription factor and tumor suppressor (29) that mediates ligand-independent apoptosis via caspase-8 activation in breast cancer cells (14). As expected, IRF1 expression was increased by IFN-α treatment in both primary KCs (**Supplementary Figure S4A**) and N/TERTs (**Figures 5A–C**). We used Accell siRNA to knockdown expression of IRF1 (siIRF1) in N/TERTs (**Figure 5D**). siIRF1 and non-targeting control N/TERTs were treated with IFN-α and UVB and caspase-3 cleavage was assessed. There was no difference in baseline levels of UV-mediated caspase-3 activation with IRF1 knockdown; however, suppression of IRF1 significantly reduced the effect of IFN-α priming to induce enhanced apoptosis (**Figures 5E, F**). This suggests that IFN induction of IRF1 is required for enhanced apoptosis following UVB exposure.

## 4 Discussion

In this paper, we identified that type I IFN priming of KCs prior to UVB exposure resulted in increased caspase-8-driven apoptosis that was independent of XAF1 and death ligands, but dependent on IRF1. Further, we observed a similar phenomenon *in vivo* in which epidermal overexpression of IFN-κ increased UVB-induced apoptosis. Dissecting these pathways provides important insights into how type I IFN production may drive sensitivity to sunlight in SLE.

Stimulation with type I IFNs has been implicated in activation of several cell death pathways. For example, type I IFN stimulation of apoptosis-resistant fibroblasts and colon adenocarcinoma cell lines induces necroptosis (30, 31). Conversely, type I IFN signaling during influenza infection of respiratory epithelial cells induces a switch from apoptosis to pyroptosis to promote pro-inflammatory responses (32). Our lab has previously shown by TUNEL that KCs from SLE patients die more after UV exposure compared to healthy controls and that this is dependent on IFN signaling (13). Here, we expand on these findings by clarifying that IFN-priming of KCs does not alter the apoptotic pathways activated by UVB exposure, just amplifies them. Further, while UVB exposure is associated with activation of both extrinsic and intrinsic pathways of apoptosis (23, 24) we demonstrate specific enhancement of caspase-8-driven apoptosis by type I IFNs. These findings contribute to our mechanistic understanding of how the inflammatory environment present even in non-lesional SLE skin can promote lesion induction and disease flares upon UV exposure.

Our data demonstrate a role for IRF1 in regulating IFN-mediated enhancement of UVB-induced apoptosis. It is well established that IRF1 regulates a large number of genes associated with apoptosis, including caspase-8 (33, 34). We have shown that IFN-treated KCs upregulate expression of caspase-8 and IRF1 and that both are required for IFN-enhancement of UVB-driven apoptosis; however, the full mechanism by which this occurs needs further investigation. It may be that increased expression of caspase-8 following type I IFN treatment sensitizes cells for activation of apoptotic signaling pathways upon a subsequent stimulus. Since we have shown this process to be independent of known death ligands, we hypothesize that UVB stimulation induces clustering of death receptors that allows for activation of caspase-8 in the absence of ligand binding. Previous work has shown that UV induces receptor clustering (35), including aggregation of Fas through reorganization of membrane lipid rafts that results in apoptosis of melanoma cells (36, 37). Whether this occurs in IFN-α primed, UVB-exposed KCs to initiate apoptosis should be examined. Of note, loss of IRF1 did not entirely abrogate apoptosis, but rather reduced the IFN effect. This suggests that IRF1 specifically drives the IFN enhancement of apoptosis rather than UV-induced apoptosis as a whole. In healthy skin, apoptosis that proceeds UV exposure generally occurs as a protective response to remove irreversibly damaged precancerous cells. Thus, the ability to modulate the rate of apoptosis in IFN-exposed cells without entirely turning it off has attractive therapeutic potential.

While a role for necroptosis in driving inflammatory responses to UV light in IFN-rich skin was ruled out in our study, nucleic acid sensor Z-DNA-binding protein 1 (ZBP1), typically recognized for its role in inflammatory cell death, may still be an important mediator of photosensitivity in SLE. Recent work identified a cell death-independent function of ZBP1 during times of mitochondrial dysfunction (38). Upon mitochondrial DNA (mtDNA) stress, Z-DNA accumulates in the mitochondria and the cytosol. ZBP1 expression is strongly induced and this promotes cGAS localization to the cytosol where these sensors interact via the RHIM domains of ZBP1 and the N-terminus of cGAS. The DNA binding domains of both ZBP1 and cGAS are bridged by DNA. This DNA binding activates the cGAS-STING pathway and subsequent type I IFN production. ZBP1 enhances the cytosolic retention of cGAS potentiating a sustained IFN response. While this study was done in fibroblasts and cardiomyocytes, ZBP1 is expressed in keratinocytes and is known to contribute to skin inflammation (39). It is well known that UVB irradiation induces mitochondrial dysfunction, therefore, future research should examine the involvement of these pathways in SLE.

Intriguingly, we identified strong upregulation of XAF1 in IFN-α-stimulated KCs; however, loss of XAF1 further enhanced rates of KC apoptosis after UVB exposure. While XAF1 has conventionally been regarded as a pro-apoptotic protein, our study is the first to investigate its role in UVB-mediated apoptosis. XAF1 can act in a variety of ways to promote apoptosis including through stabilization of p53 (40, 41). Interestingly, reduction of p53 in KCs has been shown to enhance apoptotic susceptibility of cells to UV irradiation (42) as well as to IFN treatment (43), suggesting pro-survival functions for p53. Further, studies in HT29 colon carcinoma cell lines showed that expression of p53 after UV irradiation stimulates apoptosis, while expression of p53 prior to irradiation can protect against apoptosis (44). In our study, KCs were pretreated with IFNs prior to exposure to UV, therefore, IFN-induction of XAF1 may result in p53 stabilization that turns on its pro-survival functions. This IFN-mediated stabilization of p53 through XAF1 induction, although seemingly counterintuitive, may partially explain the increase in apoptosis observed following silencing of XAF1. Further studies are needed to confirm this.

It is important to note that in our model, we used IFN-α stimulation to mimic the type I IFN-rich environment of lupus skin and to allow us to dissect the specific contributions of IFNs to cell death. We showed that this lowers the dose of UV required for apoptosis induction. However, this system does not fully recapitulate the altered immune landscape of the diseased state that results in abnormal UV responses in lupus skin. However, it is reassuring that we were able to detect enhancement of UV-mediated apoptosis in our murine model of *Ifnk* overexpression. Other limitations of our study include the use of caspase inhibitors. While AV/PI staining showed less complete inhibition of cell death with IETD (caspase-8 inhibitor) compared to zVAD (pan-caspase inhibitor) treatment, the more complete inhibition of caspase-3 activation by IETD suggests that caspase-8 inhibition in our experimental setting may drive some amount of compensatory non-caspase-3-dependent cell death that is identified only by AV/PI staining and blocked by zVAD. Further, the sensitivity of caspase inhibitors for specific caspases can be limited in the presence of multiple caspases (45), thus, highlighting the importance of including parallel genetic approaches, which in our case confirmed the inhibitor data.

In conclusion, our data contribute to a more thorough understanding of the mechanisms by which the type I IFN-rich environment of lupus skin contributes to aberrant responses to UVB. This opens new avenues for potential therapeutic options for patients whose disease is flared by the sun.

## Data availability statement

Publicly available datasets were analyzed in this study. This data can be found here: Gene Expression Omnibus (https://www.ncbi.nlm.nih.gov/geo/query/acc.cgi?acc=GSE124939) under accession number GSE124939.

## Ethics statement

The studies involving humans were approved by University of Michigan Institutional Review Board. The studies were conducted in accordance with the local legislation and institutional requirements. The participants provided their written informed consent to participate in this study. The animal study was approved by The University of Michigan Institutional Animal Care and Use Committee. The study was conducted in accordance with the local legislation and institutional requirements.

## Author contributions

SL: Conceptualization, Formal analysis, Funding acquisition, Investigation, Methodology, Writing – original draft, Writing – review & editing. MG-K: Investigation, Writing – original draft, Writing – review & editing. BX: Investigation, Resources, Writing – original draft, Writing – review & editing. TM: Investigation, Writing – original draft, Writing – review & editing. AH: Investigation, Writing – original draft, Writing – review & editing. MM: Investigation, Writing – original draft, Writing – review & editing. BK: Investigation, Writing – original draft, Writing – review & editing. JG: Resources, Writing – original draft, Writing – review & editing. JK: Conceptualization, Formal analysis, Funding acquisition, Methodology, Project administration, Resources, Supervision, Writing – original draft, Writing – review & editing.
