# Supplementary material for: Interferon alpha promotes caspase-8 dependent ultraviolet light-mediated keratinocyte apoptosis via interferon regulatory factor 1
Source: Front Immunol. 2024 Apr 10;15:1384606. doi: 10.3389/fimmu.2024.1384606 (PMC11039837; doi:10.3389/fimmu.2024.1384606)
Supplement: Supplementary file 1 [file DataSheet_1.pdf]

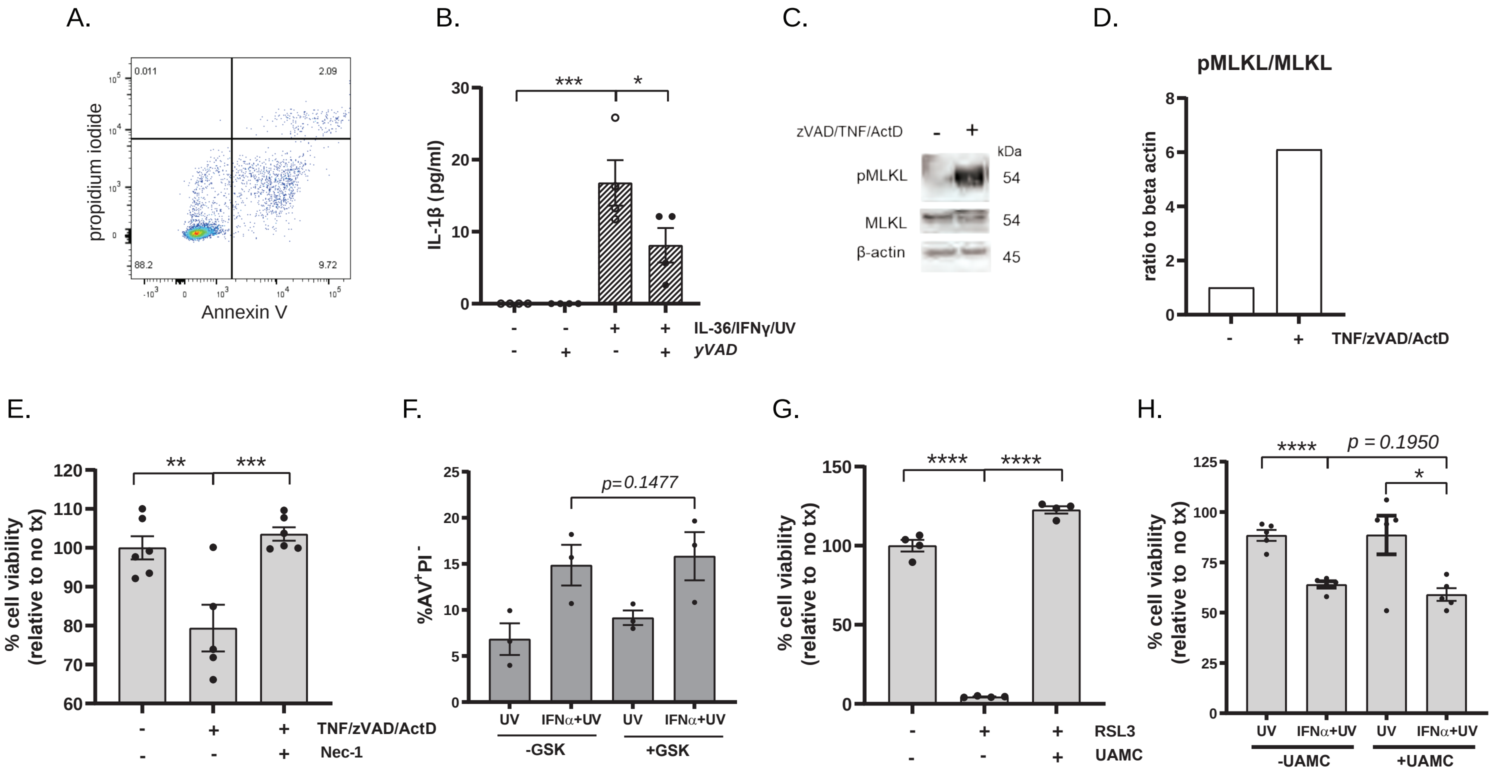

Supplementary Figure 1. (A) Representative flow cytometry gating strategy. Debris was gated out using FSC/SSC and single cells were assessed for Annexin V (AV) and propidium iodide (PI). **(B)** IL-1β release was assessed by ELISA in the supernatant of N/TERTs treated with or without 10μM Ac-YVAD-cmk (yVAD) for 30 minutes, 25 ng/ml IL-36/IFNγ for 24 hours, then exposed to 50mJ/cm² UVB (*n*=4). **(C)** Representative Western blot and **(D)** quantification (shown as a ratio) of pMLKL and MLKL in N/TERTs treated with or without 20μM Z-VAD-FMK (zVAD) for 30 minutes and 20ng/ml TNF-α and 1μg/ml Actinomycin D (ActD) for four hours. **(E)** Viability of N/TERTs treated with or without 10μM Necrostatin-1 (Nec-1) and/or 20μM Z-VAD-FMK (zVAD) for 30 minutes followed by treatment with or without 20ng/ml TNF-α and 1μg/ml ActD for four hours as measured using the Cell Counting Kit (*n*=5-6). **(F)** N/TERTs pretreated with or without 10μM RIPK3-inhibitor GSK'872 (GSK), treated with 1000U/ml IFN-α for 16 hours, then exposed to 50mJ/cm² UVB. Cell death response assessed four hours following UVB exposure by AV/PI staining and flow cytometry (*n*=3). **(G, H)** Viability of N/TERTs treated with or without 1μM UAMC-3203 (UAMC) for one hour prior to treatment with **(G)** 1μM RSL3 for 16 hours or **(H)** 1000U/ml IFN-α for 16 hours and 50mJ/cm² UVB for four hours as measured by the Cell Counting Kit (*n*=5). Data analyzed by (B, E, G) Ordinary one-way ANOVA and (F, H) paired or unpaired *t* tests. *<0.05, **<0.01, ***<0.001, ****<0.0001.

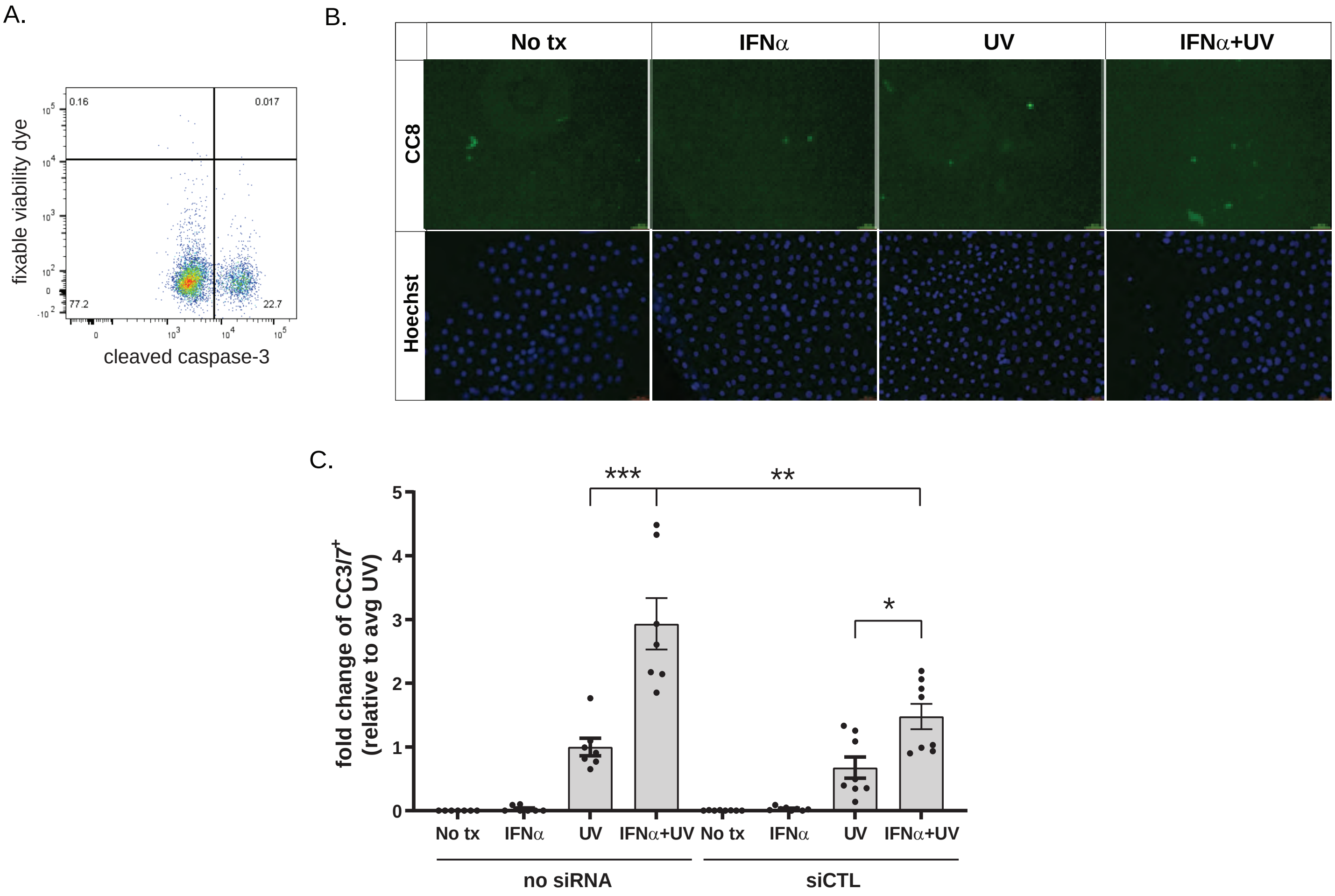

Supplementary Figure 2. (A) Representative flow cytometry gating strategy. Debris was gated out using FSC/SSC and single cells were assessed for cleaved caspase-3 (CC3) and fixable viability dye. **(B)** Representative images of cleaved caspase-8 (CC8; green) expression in N/TERTs treated with or without 1000U/ml IFN-α for 16 hours, then exposed to 50mJ/cm² UVB. Scale bar: 50μm. **(C)** Quantification of cleaved caspase-3/7 in N/TERTs incubated without (no siRNA) or with 1μM scrambled siRNA (siCTL) then treated with or without IFN-α and UVB as before (*n*=7-8). Data analyzed by unpaired *t* test or Mann-Whitney test. *<0.05, **<0.01, ***<0.001.

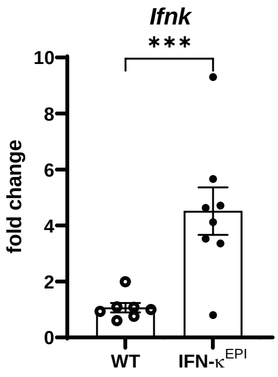

Supplementary Figure 3. Fold change expression of *Ifnk* in wild-type (WT) C57BL/6 and IFN-κ^{EPI} mice skin at baseline, assessed by qRT-PCR (*n*=7-8). Data analyzed by unpaired *t* test. ***<0.001.

A.

Gene Name	Protein Name	Healthy Control + IFN-α		SLE + IFN-α	
		log2 FC	*p*-value	log2 FC	*p*-value
XAF1	XIAP Associated Factor 1 (XAF1)	4.03	0.051	4.79	**2.01E-06**
TNFSF10	TNF-Related Apoptosis-Inducing Ligand (TRAIL)	4.08	**2.93E-11**	4.37	**7.79E-27**
TNF	TNF-alpha (TNF-α)	0.37	0.075	0.37	**0.046**
TNFRSF1B	TNF-Receptor 2 (TNF-R2)	1.36	**0.007**	1.8	**2.04E-06**
TNFSF12	TNF-Related Weak Inducer of Apoptosis (TWEAK)	0.04	0.871	0.21	0.299
TNFRSF12A	Fn14	-0.1	0.375	0.18	**0.001**
FAS	Fas	0.18	0.167	0.27	**0.0004**
FASLG	Fas Ligand (FasL)	nd	--	nd	--
IRF1	Interferon Regulatory Factor 1 (IRF1)	1.66	**5.26E-06**	2.09	**3.95E-07**
CASP8	Caspase-8	1.08	**1.37E-13**	0.83	**1.20E-26**

B.

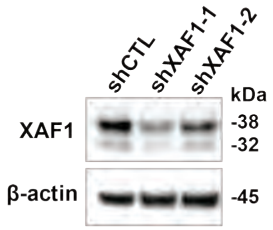

C.

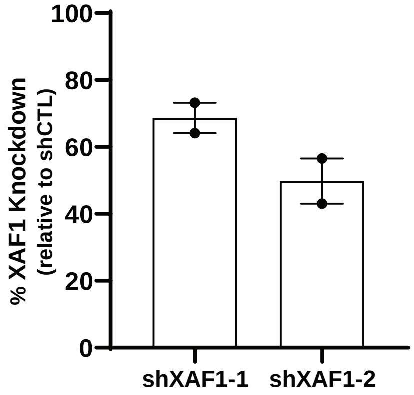

Supplementary Figure 4. (A) Primary keratinocytes were isolated from non-lesional SLE skin and healthy control skin, treated with or without 1000U/ml IFN-α for six hours, and gene expression was analyzed by RNA-sequencing as previously reported (17). Gene expression values are represented as log2 fold change (FC) and *p*-values <0.05 were considered significant (nd, not detected). **(B, C)** Stable XAF1-knockdown N/TERT lines were generated by transducing N/TERTs with lentivirus expressing either of two shRNAs targeting XAF1 (shXAF1-1, -2) or a control shRNA (shCTL). Protein knockdown was confirmed by Western blot (*n*=2).

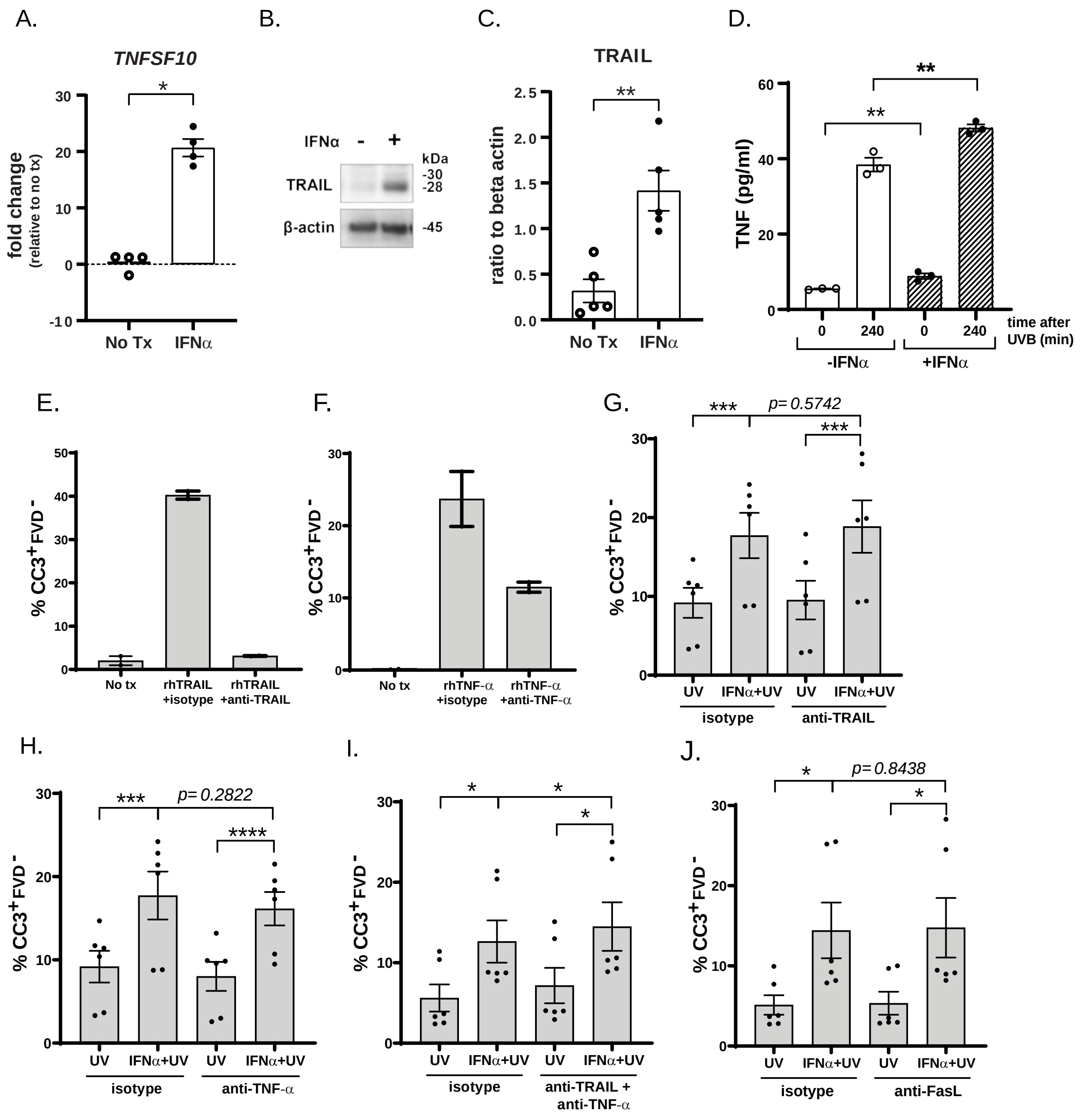

Supplementary Figure 5. N/TERTs were treated with or without 1000U/ml IFN-α for 16 hours and **(A)** fold change expression of *TNFSF10* was assessed by qRT-PCR (*n*=4) and **(B, C)** protein levels of TRAIL were assessed by Western blot (*n*=5). **(D)** TNF-α release was assessed by ELISA in the supernatant of N/TERTs treated with IFN-α as before and exposed to 50mJ/cm² UVB (*n*=3). **(E)** N/TERTs were treated with 100ng/ml anti-TRAIL or isotype control and 50ng/ml recombinant human (rh) TRAIL for 24 hours then cleaved caspase-3 (CC3) and fixable viability dye (FVD) staining was assessed by flow cytometry (*n*=2). **(F)** Primary human fibroblasts were treated with 5µg/ml anti-TNF-α, 1µg/ml actinomycin D, and 0.75ng/ml rhTNF-α for 24 hours then CC3 and FVD staining was assessed by flow cytometry (*n*=2). **(G-J)** N/TERTs were treated with or without IFN-α and UVB as before. Immediately following UVB exposure cells were treated with neutralizing antibodies targeting **(G)** TRAIL (100ng/ml), **(H)** TNF-α (5µg/ml), **(I)** TRAIL+TNF-α, or **(J)** FasL (1µg/ml) or isotype controls. Four hours later, CC3 and FVD staining was assessed by flow cytometry (*n*=6). Data analyzed by paired or unpaired *t* test for normally distributed data and Wilcoxon matched-pairs signed rank test or Mann-Whitney test for non-normally distributed data. * <0.05, **<0.01, ***<0.001.
